# Supplementary material for: Pan-Canadian Analysis of Practice Patterns in Small Cell Carcinoma of the Cervix: Insights from a Multidisciplinary Survey
Source: Curr Oncol. 2024 May 3;31(5):2610–9. doi: 10.3390/curroncol31050196 (PMC11119600; doi:10.3390/curroncol31050196)

Figure S2: Practice patterns in the treatment of SCNECC with local relapse (A) and regional nodal relapse (B). Abbreviations: NAC (neoadjuvant chemotherapy), + (followed by), C (chemotherapy), RT (radiotherapy)

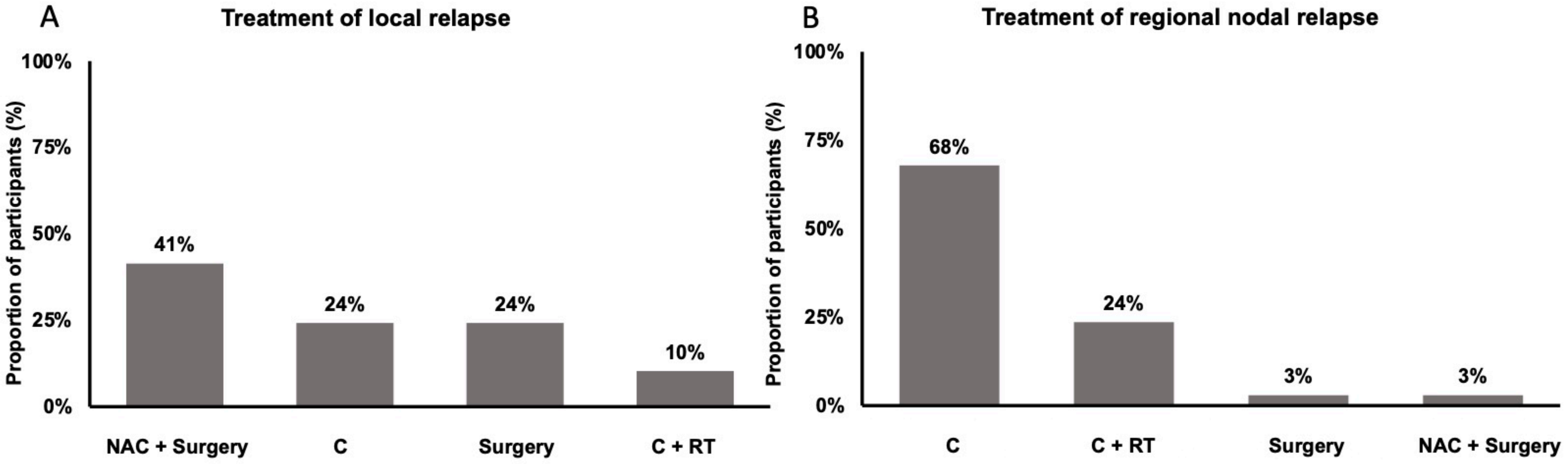

Supplement: Supplementary file 1 [file curroncol-31-00196-s001.zip › Figure S2 Jan 20.pdf]
