# Supplementary material for: Pan-Canadian Analysis of Practice Patterns in Small Cell Carcinoma of the Cervix: Insights from a Multidisciplinary Survey
Source: Curr Oncol. 2024 May 3;31(5):2610–9. doi: 10.3390/curroncol31050196 (PMC11119600; doi:10.3390/curroncol31050196)

Figure S3: Practice patterns in the surveillance of patients with SCNECC after curative intent treatment. Abbreviations: H+P (history and physical examination), q (every), CT (CT chest, abdomen and pelvis), PET (18-FDG-PET/CT; positron emission tomography), MRI (magnetic resonance imaging)

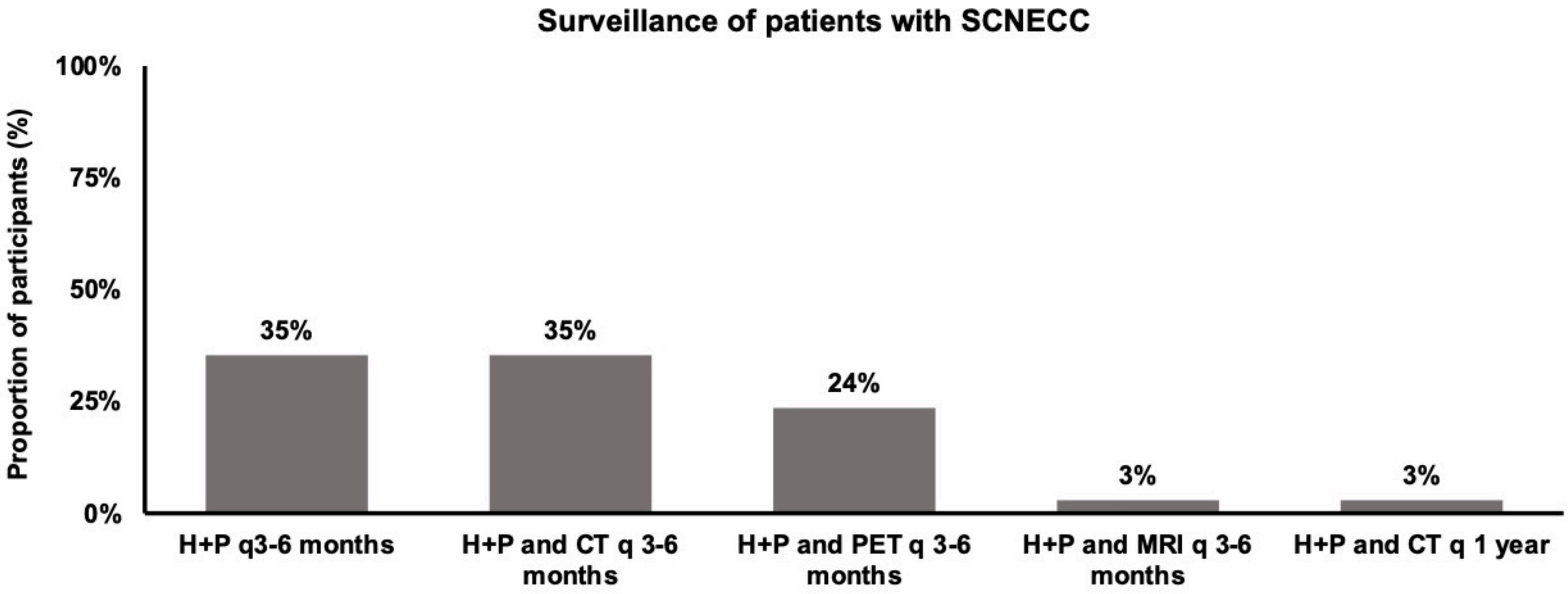

Supplement: Supplementary file 1 [file curroncol-31-00196-s001.zip › Figure S3 Jan 20.pdf]
