# Supplementary material for: Pan-Canadian Analysis of Practice Patterns in Small Cell Carcinoma of the Cervix: Insights from a Multidisciplinary Survey
Source: Curr Oncol. 2024 May 3;31(5):2610–9. doi: 10.3390/curroncol31050196 (PMC11119600; doi:10.3390/curroncol31050196)
Supplement: Supplementary file 1 [file curroncol-31-00196-s001.zip › S4 SCNECC Survey.pdf]

Canadian survey of practise patterns in the treatment of small cell carcinoma of the cervix

Thank you for participating in this anonymous survey on practise patterns in the treatment of small cell carcinoma of the cervix (SCCC) across Canada. This survey has 23 questions and takes approximately 10 minutes to complete.

* 1. On average, how many patients with small cell carcinoma of the cervix (SCCC) are treated annually at your centre?

- ◯ 0
- ◯ 1-4
- ◯ 5-10
- ◯ >10

* 2. Please confirm your state/province of practice

[ ]

3. Please enter the name of your primary centre of practice (optional)

[ ]

* 4. Do practice at an academic institution?

- ◯ Yes
- ◯ No

* 5. Does your centre have the following services (please check all that apply)?

- ☐ Gynecologic oncologic surgery sub-specialty services
- ☐ Radiation oncology services
- ☐ Medical oncology services

* 6. At your centre, primarily which specialty delivers systemic therapy for SCCC?

- ◯ Gynecologic Oncologist
- ◯ Radiation Oncologist
- ◯ Gyne Medical Oncologist
- ◯ Other Medical Oncologist

* 7. What is your primary specialty?

- ○ Gynecologic Oncology
- ○ Medical Oncology
- ○ Radiation Oncology

Other (please specify)

[ ]

* 8. For how many years have you been in practice?

- ○ ≤5
- ○ 6-10
- ○ 11-15
- ○ ≥16

* 9. At your centre, what clinical and imaging modalities do you use for staging SCCC patients? (select up to 3)

- ☐ MRI pelvis
- ☐ CT scan (chest abdomen pelvis)
- ☐ CT pelvis only
- ☐ PET
- ☐ EUA with sigmoidoscopy and cystoscopy
- ☐ None

Other (please specify)

[ ]

* 10. Do you routinely obtain brain imaging?

- ○ For everyone
- ○ Only if symptoms
- ○ Only if lung mets
- ○ Only if symptoms or lung mets

* 11. At your centre which molecular markers are typically obtained? (select all that apply)

- [ ] P16
- [ ] HPV
- [ ] Chromogranin
- [ ] Synaptophysin
- [ ] P53
- [ ] PDL1
- [ ] MMR
- [ ] NGS panel
- [ ] NSE
- [ ] CD56

Other (please specify)

* 12. What is the typical treatment regimen at your Centre for patients with early stage SCCC (stage 1A1-1B2)?

	Most commonly used regimen	Alternative regimen	Another alternative regimen
Surgical resection only	◯	◯	◯
Surgery followed by adjuvant chemotherapy	◯	◯	◯
Neoadjuvant chemotherapy followed by surgical resection only	◯	◯	◯
Neoadjuvant chemotherapy followed by surgery ± radiation (EBRT ± brachytherapy)	◯	◯	◯
Neoadjuvant chemotherapy followed by radiation (EBRT ± brachytherapy)	◯	◯	◯
Concurrent chemoradiotherapy (+ brachytherapy) only	◯	◯	◯
Concurrent chemoradiotherapy (+ brachytherapy) followed by adjuvant chemotherapy	◯	◯	◯
N/A (select this if you rarely use an alternative regimen)	◯	◯	◯
N/A (select this if you rarely use an alternative regimen)	◯	◯	◯

Other (please specify)

[ ]

* 13. What is the typical treatment regimen at your centre for patients with locally advanced nonmetastatic SCCC (stage 1B3-IVA)?

	Most commonly used regimen	Alternative regimen	Another alternative regimen
Surgical resection only	○	○	○
Surgery followed by adjuvant chemotherapy	○	○	○
Neoadjuvant chemotherapy followed by surgical resection only	○	○	○
Neoadjuvant chemotherapy followed by surgery ± radiation (EBRT ± brachytherapy)	○	○	○
Neoadjuvant chemotherapy followed by radiation (EBRT ± brachytherapy)	○	○	○
Concurrent chemoradiotherapy (+ brachytherapy) only	○	○	○
Concurrent chemoradiotherapy (+ brachytherapy) followed by adjuvant chemotherapy	○	○	○
N/A (select this if you rarely use an alternative regimen)	○	○	○
N/A (select this if you rarely use an alternative regimen)	○	○	○

Other (please specify)

[ ]

* 14. What chemotherapy regimen do you typically prescribe when treating concurrently with radiotherapy?

- ○ Cisplatin/Carboplatin + Etoposide
- ○ Single agent platinum
- ○ Cisplatin/Carboplatin + Paclitaxel
- ○ Cisplatin + Irinotecan
- ○ Other (please specify)

[ ]

15. Do you offer additional chemotherapy in the frontline treatment after concurrent chemoRT? If so, please specify which chemotherapy regimen?

- ◯ Cisplatin/Carboplatin + Etoposide
- ◯ Single agent platinum
- ◯ Cisplatin/Carboplatin + Paclitaxel
- ◯ Other (please specify)
- ◯ Cisplatin + Irinotecan
- ◯ Typically do not offer additional chemotherapy after chemoRT

16. Do you offer additional chemotherapy in the frontline treatment after concurrent chemoRT? If so, please specify how many cycles?

- ◯ 2
- ◯ 4
- ◯ 6
- ◯ >6
- ◯ Typically do not offer additional chemotherapy after chemoRT

17. When prescribing radiation therapy for the curative treatment of SCCC, what dose fraction do you typically use?

- ◯ 30Gy in 10 fractions
- ◯ 40Gy in 15 fractions
- ◯ 45Gy in 25 fractions only
- ◯ 45Gy in 25 fractions + brachytherapy
- ◯ 50.8Gy in 28 fractions only
- ◯ 50.8Gy in 28 fractions + brachytherapy
- ◯ We do not treat SCCC with radiation therapy at our centre
- ◯ Other (please specify)

* 18. For a patient with local relapse after curative chemoradiation, what treatment is typically used?

- ◯ Surgery only
- ◯ Chemotherapy plus surgery
- ◯ Chemotherapy plus radiation
- ◯ Chemotherapy only
- ◯ Radiation
- ◯ None
- ◯ Other (please specify)

* 19. For a patient with regional nodal relapse after curative chemoradiation, what treatment is typically used?

- ◯ Surgery only
- ◯ Chemotherapy plus surgery
- ◯ Chemotherapy plus radiation
- ◯ Chemotherapy only
- ◯ Radiation
- ◯ None
- ◯ Other (please specify)

* 20. What systemic therapy regimen would you use for a patient with distant relapse? (Rank as 1st line, 2nd line, 3rd line, etc.).

	1st line	2nd line	3rd line
Cisplatin/carboplatin + etoposide	◯	◯	◯
Cisplatin/carboplatin + paclitaxel	◯	◯	◯
Paclitaxel + topotecan + bevacizumab	◯	◯	◯
Vincristine + doxorubicin + cyclophosphamide	◯	◯	◯
Single agent platinum	◯	◯	◯
Immunotherapy	◯	◯	◯
Cisplatin/carboplatin + paclitaxel + bevacizumab + pembrolizumab	◯	◯	◯
Clinical trial	◯	◯	◯
Best supportive care	◯	◯	◯
I Don't Know	◯	◯	◯

Other (please specify)

* 21. What systemic therapy regimen would you use for a patient with newly diagnosed metastatic (stage IVB) SCCC? (Rank as 1st line, 2nd line, 3rd line, etc.)

	1st line	2nd line	3rd line
Cisplatin/carboplatin + etoposide	○	○	○
Cisplatin/carboplatin + paclitaxel	○	○	○
Paclitaxel + topotecan + bevacizumab	○	○	○
Vincristine + doxorubicin + cyclophosphamide	○	○	○
Single agent platinum	○	○	○
Immunotherapy	○	○	○
Cisplatin/carboplatin + paclitaxel + bevacizumab + pembrolizumab	○	○	○
Clinical trial	○	○	○
Best supportive care	○	○	○
I don't Know	○	○	○

Other (please specify)

* 22. After curative intent treatment of limited stage SCCC, what surveillance regimen do you typically choose?

- ○ History and physical (H+P) q3-6 months
- ○ H+P q 1 year
- ○ H+P and CT q 3-6 months
- ○ H+P and CT 1 year
- ○ H+P and PET q 3-6 months
- ○ H+P and PET 1 year
- ○ Other (please specify)

* 23. Do you consent to your anonymous responses being used for research purposes?

- ○ Yes
- ○ No
